# The role of monitoring and evaluation to ensure functional access to community-based early diagnosis and treatment in a malaria elimination programme in Eastern Myanmar

**DOI:** 10.1186/s12936-019-2677-2

**Published:** 2019-02-22

**Authors:** Jade D. Rae, Suphak Nosten, Stéphane Proux, Aung Myint Thu, Win Cho Cho, K’Nyaw Paw, Eh Shee Paw, Paw Bway Shee, Saw Aye Be, Saw Hsa Dah, Saw Ku Ler Moo, Saw Myo Chit Minh, Paw Wah Shee, Jacher Wiladphaingern, Saw Win Tun, Ladda Kajeechiwa, May Myo Thwin, Gilles Delmas, François H. Nosten, Jordi Landier

**Affiliations:** 1 Shoklo Malaria Research Unit, Mahidol-Oxford Tropical Medicine Research Unit, Faculty of Tropical Medicine, Mahidol University, Mae Sot, Thailand; 2 Centre for Tropical Medicine and Global Health, Nuffield Department of Medicine, University of Oxford, Oxford, UK; 3 IRD, Aix Marseille Université, INSERM, SESSTIM, Sciences Economiques & Sociales de la Santé & Traitement de l’Information, Médicale, Marseille, France

**Keywords:** Malaria elimination, Monitoring and evaluation, Surveillance, Malaria post

## Abstract

**Background:**

Improving access to early diagnosis and treatment (EDT) has increasingly proven to be a major contributor to decreasing malaria incidence in low-transmission settings. The Malaria Elimination Task Force (METF) has deployed malaria posts set up in Eastern Myanmar, providing free uninterrupted community-based access to EDT in more than 1200 villages. Ensuring high quality services are provided by these malaria posts is essential to reaching elimination targets. The present study aimed to determine the functionality of the malaria posts in the METF programme.

**Methods:**

This report analysed routinely collected data (weekly reports, individual consultation, diagnostic test quality control) and data collected specifically during monitoring and evaluation visits using descriptive statistics and univariate logistic regression. The presence of major dysfunctions (stock-outs and reported closing; likely to impair the ability of the population to access EDT) or minor dysfunctions (no formal METF training, lack of regular salary, forms and manual not on-site, and low frequency of supervisor visits) and the ability to anticipate dysfunctions through analysis of weekly reports were assessed.

**Results:**

A total of 65% of malaria posts had no major dysfunction identified during monitoring and evaluation visits, while 86% of malaria posts were fully stocked with tests and medicines used for treatment. Diagnosis was correctly conducted with few false positives and rare mis-speciation of results. Malaria post worker knowledge of malaria treatments showed few gaps, mostly in the treatment of more complex presentations. Malaria posts were well utilized in the population, with 94% of consultations occurring within the first 3 days of fever. In the regression analysis, reported stock-outs and delayed weekly reports were associated with observed major and minor dysfunctions in monitoring and evaluation visits, emphasizing the need to reinforce support to malaria post supervisors, who were responsible for the local logistics of supply and data transmission and day-to-day supervision.

**Conclusion:**

The malaria posts operating under the METF programme perform to a high standard, with the majority offering uninterrupted access to diagnosis and treatment, and high service uptake in the villages serviced by the programme. However, programme operations can be strengthened by increasing malaria post supervisor visits and re-training malaria post workers.

**Electronic supplementary material:**

The online version of this article (10.1186/s12936-019-2677-2) contains supplementary material, which is available to authorized users.

## Background

In countries where malaria remains endemic the increased funding support and strengthening of local health system has enabled the successful deployment of vector control measures and the improvement of case detection and management [1]. These efforts have resulted in substantial reductions in the number of malaria cases and deaths since 2010, many of these reductions occurring in the South-East Asia region [1], where early detection and treatment of malaria is a cornerstone of elimination programmes. These progresses are under the threat of multidrug resistant parasites [2, 3]. Furthermore, weakening of control programmes leading to local interruption of service delivery is a major factor of malaria resurgence [4]. Vigilance must be maintained in order to reach elimination goals.

Provision of uninterrupted access to early diagnosis and effective malaria treatment to all individuals living in endemic regions is a necessity to control morbidity and mortality, but also to successfully reach elimination targets [5]. This remains a challenge in regions where political, geographic, economic, and socio-cultural barriers exist [6–9].

### The malaria post

Many barriers can be lifted through basic village-based malaria clinics, or “malaria posts” (MP) operated by trained members of the community. The malaria post workers (MPWs) are trained in basic malaria biology and ecology, how to prevent malaria, how to use rapid diagnostic tests (RDTs) to diagnose malaria, and how to administer artemisinin-based combination therapy (ACT) or other required treatments after parasitological confirmation.

A functional MP is defined as a free uninterrupted access point to reliable diagnosis and effective treatment of clinical malaria within 24–48 h of fever onset, operated by a trained MPW, and supervised regularly to ensure a high quality of service.

A functional MP can contribute significantly to the local reduction of malaria transmission [10] and programmes establishing region-wide networks of community-based access to malaria treatment in various settings have described a decrease in all-cause mortality including malaria and improved additional health outcomes [11, 12].

### Monitoring and evaluation

The funding of malaria elimination programmes is essential for their operation and programmes must provide evidence for effective performance and impact. In addition, epidemiological surveillance is a critical component of elimination. Strong information systems for data collection must, therefore, be in place [13], with this data used to improve and inform programme operations.

Essential to the role of malaria post workers (MPWs) is the keeping of accurate records of RDT use and results, ACT use, and reporting stocks of RDTs and ACT medicines. This information provides feedback to programme managers on MP activities, supplies, and epidemiological trends, which are of importance in ensuring effective MP functioning.

Monitoring and evaluation (M&E) activities are able to measure service uptake, diagnosis and treatment availability, and MPW performance and knowledge. M&E reports represent a valuable data resource for measuring programme performance and impact [9] as well as being used as a tool for further intervention [14].

### Community-based early diagnosis and treatment within a regional malaria elimination programme in Eastern Myanmar

Since April 2014, the Malaria Elimination Task Force (METF) has deployed community-based access to early diagnosis and treatment at a regional level in Eastern Karen/Kayin State of Myanmar, operating more than 1200 malaria posts for a target population of 365,000 persons [15, 16]. The deployment of MPs was a key component in the strong decrease in malaria incidence recorded within the first 3 years of the programme [10].

In this article, data collected routinely and during specific M&E visits of MPs is analysed to determine how effectively MPs met the functionality criteria when delivering access to diagnosis and treatment in this region.

## Methods

### Study design and study population

**Fig. 1:**
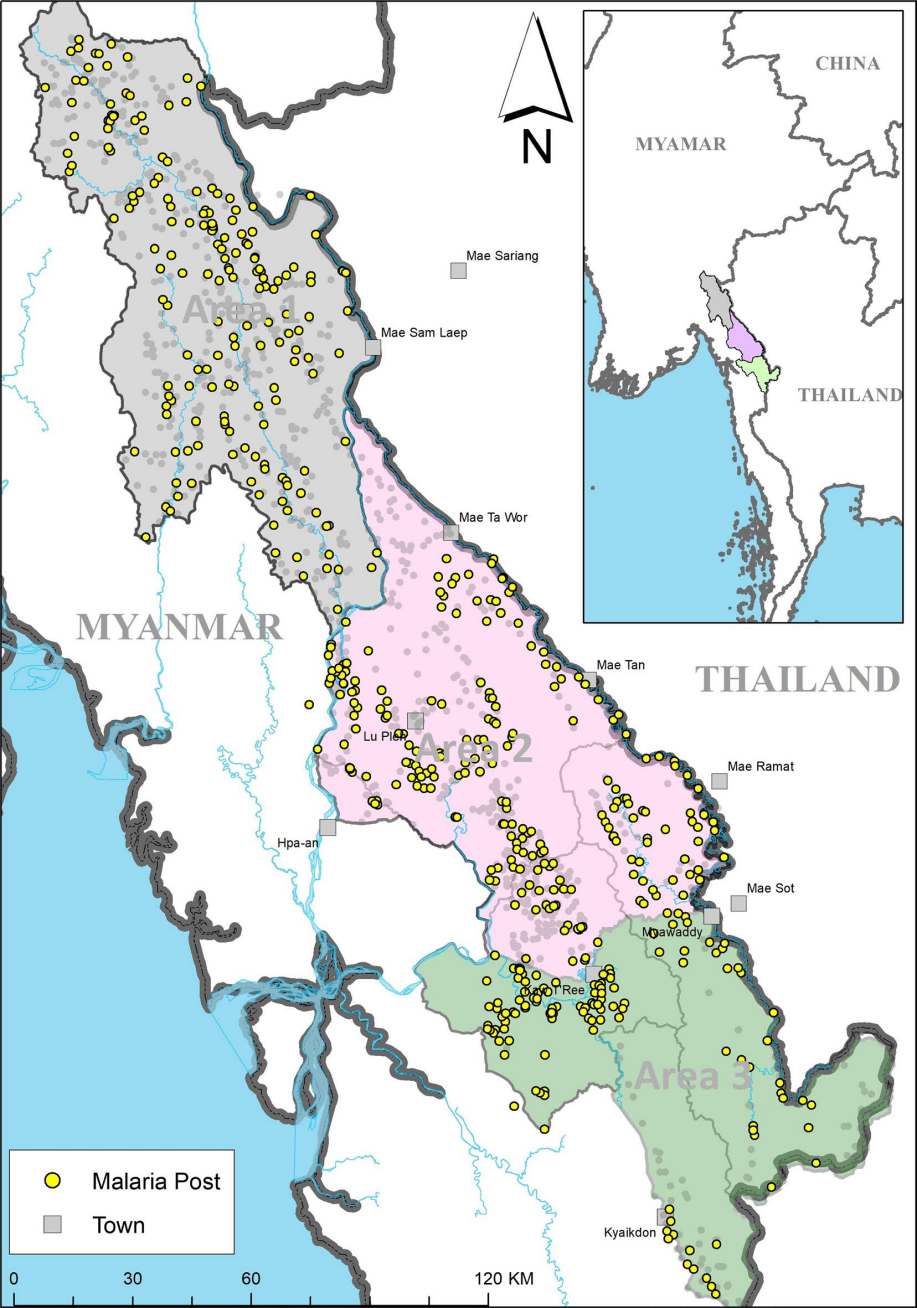
METF malaria posts that underwent monitoring and evaluation in the Karen/Kayin state of Myanmar. Area 1, in grey, is the least economically developed and consists of mountainous forest regions making travel and transportation challenging. Area 2, in pink, is divided by the Dawna range and consists both of sparsely distributed villages and more densely populated areas around cities of Hpa’an and Hlaingbwe. Similarly, Area 3, in green, consists of sparsely distributed villages divided by forests and mountains with more densely populated areas near Myawaddy and Kawkareik [16]. The yellow circles represent the villages where malaria posts have been visited during M&E, the grey circles represent unvisited malaria posts, and the grey squares represent other villages

The METF network consists of 1227 MPs spanning a geographic area of approximately 18,000 km^2^ in the Karen/Kayin State of Eastern Myanmar (Fig. 1). This region is divided into three areas (Area 1 = 482 MPs; Area 2 = 568 MPs; Area 3 = 172 MPs), where each area differs geographically and demographically, resulting in differences in ease of navigation between villages.

Each area is divided into zones, where each zone is administered by the non-governmental organization or community-based organization providing health services locally and is overseen by a zone-coordinator employed by METF [16]. A MP supervisor is assigned to 10–15 MPs in neighbouring villages. The supervisor is responsible of maintaining stocks of ACT medicines and RDTs and reporting of data to the METF central office in Mae Sot, as well as providing support to their MPWs. Typically, each MP is operated by one MPW when in a community below 50 households, and two when above.

### M&E data collection

#### Selection of malaria posts for M&E visits

M&E visits were conducted by a central team to obtain information on MP activity and functioning. These visits were conducted in MPs selected either randomly (77.54%) or targeted (20.29%) without notice given, using a set of a priori defined indicators calculated from the weekly data reports. These indicators aimed to provide an alert of potential dysfunctions in MP performance (Table 1). Each month, the weekly data was analysed, generating a list of the MPs with the highest number of alerts for the previous month of reporting. The number of MPs selected for visitation each month by M&E was based on season and geographical location. In the wet season, defined as May–October in this region, the number of visits possible was reduced (20 in area 1, 40 in areas 2 and 3) due to environmental factors making it harder to navigate. In the dry season, defined as November–April, the number of possible visits was higher (30 in area 1, 60 in areas 2 and 3).

**Table 1:** Definition of alerts defined to identify malaria posts selected for monitoring and evaluation

Alert	Definition
Late report	More than 21 days for SMS reports, more than 28 days for paper reports from start of reporting week
Missing report	No report for this week
Report 0 RDT	Reported 0 stock for RDTs
Report 0 ACT	Reported 0 stock for ACT medicines
Correct RDT use	Each febrile patient at MP has been tested using RDT; measured as weekly total consultations = weekly total number of valid RDT
Correct treatment use	Each febrile patient with a positive RDT diagnosis is treated; measured as weekly total malaria positive RDT = weekly total number of febrile patients treated^a^
Invalid RDTs repeated	All invalid RDT results are repeated; measured as total number of valid RDTs < than the total number of consultations and an invalid RDT reported

^a^This definition is too stringent as febrile patients with a *P falciparum* positive RDT were not treated if treatment had been administered within the last 14 days as HRP2 can remain in the blood for 2 weeks following treatment and parasite clearance, leading to a possible false positive RDT

A pilot phase was conducted in a small number (2.17%) of MP in August 2016, followed by random visits between September 2016 and May 2017. Targeted visits began in June 2017 with each list of MPs for M&E containing half selected MPs based on alerts, and half random MPs out of those not yet visited.

#### Monitoring and evaluation data

Information collected by the M&E team consisted of MP stock management, training, supervisor visits, salary received, MP closure and whether there was another MP in the area operated by another medical structure (see data collection sheet in Additional file 1). The M&E team also checked stocks of ACT medicines and RDTs and ensured the information in the consultation sheet was recorded correctly.

A questionnaire on malaria treatment was included in M&E visits from January 2017 to gain information on knowledge of correct malaria treatment guidelines, Additional file 2 shows the questionnaire used (see treatment questionnaire in Additional file 2). The questionnaire was administered to MPWs during M&E visits but also opportunistically on other occasions such as at training sessions and meetings where MP workers were available. On these occasions, non-MP worker METF staff with higher level medical training (MP supervisors and zone coordinators) were also asked to complete the questionnaire.

### Definitions of MP functionality

Based on information collected during the M&E visits, a MP was defined as having a major dysfunction at the time of M&E visit if they reported closing for more than 24 h in the past 2 months, or if they did not have ACT medicines and RDTs in stock at the time of visit, or if they reported a stockout for more than 2 days in the previous 2 months. This definition was deliberately stringent, since ensuring no MP closure for > 2 months was an important challenge.

A minor dysfunction, unlikely to disable the MPs ability to test and treat all malaria cases, was defined as the MP being operated by an MPW who did not receive the “official” METF training, no MP supervisor visits in the previous 2 months, forms not onsite, manual not onsite, and regular salary not received. A MPW who had not receive formal METF training was considered a minor dysfunction because these MPWs were likely to have received training by their MP supervisor or, if applicable, from the MPW who they are taking over the role from. Moreover, no visit from a MP supervisor in the previous 2 months was considered only a minor dysfunction since weekly contact was maintained for data transmission: no visit did not mean no contact had occurred, but rather no contact had occurred within the MP itself.

### Variables and data sources

#### Weekly data

MP activity data was collected weekly and was used to assess MP impact and performance by measuring the number tested, the number treated, the age group of patients, supplies of ACT medicines and RDTs, village size, geographical coordinates of the MP, and the date this information was received by the data centre.

Every week, all MPs generated an activity report summarizing the number of fever consultations and their results in terms of malaria diagnosis and treatment administered, as well as stock levels [10, 15]. The MPW transmitted the report to his/her MP supervisor in paper format. The supervisor transmitted the data to the METF central office via SMS using a smartphone and a dedicated data-entry application in areas covered by the global system for mobile communications (GSM). In areas without GSM, runners collected the weekly forms from the MP supervisors and delivered them to the nearest location for online data entry [15, 16].

#### Individual consultation data

The MPW recorded on a consultation sheet the following set of basic information for each patient: date, name, age, sex, address, days since the beginning of symptoms, RDT result, treatment provided, and if necessary, other action taken (referral to hospital). The data recorded on the consultation sheet was aggregated to fill the weekly data report. The consultation sheet was transmitted monthly to be entered in a central database. The delay between the beginning of symptoms and consultation at a MP were extracted for all patients in this database for analysis.

#### RDT quality control

Every month, 20 MPs were chosen randomly to undergo RDT quality control assessment. From the chosen MPs, all RDT performed in the previous month were rechecked by the head of laboratory at SMRU in Mae Sot. First, the validity of the test was verified by assessing adequate migration of blood, clearance of background, and presence of the control line.

The reading time was analysed to ensure compliance with instructions to read the test when the background has cleared within a maximum of 30 min. Additional observable defaults, including dried blood on the side of the blood well, dirty RDT, and scratched pad were also recorded. Each line (control, *Plasmodium vivax* and *Plasmodium falciparum* test lines) was graded rechecked mainly as negative, positive, or cannot recheck when it could not be rechecked because of blood back flow. An RDT was deemed invalid when the control line was not visible with no obvious back flow or fading issues present for positive and negative RDTs.

### Statistical methods

RDT quality control data was entered and analysed with IBM SPSS Statistics v.23. All other data was entered either via the dedicated smartphone application or on a Voozanoo online database, then aggregated and was analysed using STATA v13.0 and 14.1 (StataCorp, TX USA). Univariate logistic regression was used to investigate possible associations between performance indicators in M&E data with alerts used to target M&E visits in the weekly collected data. Univariate logistic regression was also used to investigate associations between M&E results and whether M&E visit was targeted or random.

## Results

### Monitoring and evaluation visits

From August 2016 to August 2017, 552 MP operated by 755 MPWs were visited by the M&E team. From them, 12 were visited during pilot visits in August 2016, 428 were selected at random, and 112 were selected based on predefined alerts generated from weekly data reports.

From area 1 a total of 39.83% (192/482), from area 2 a total of 39.61% (225/568), and from area 3, 76.27% (135/177) of MPs were visited. There were 2 MPWs in 4.69% (9/192) villages of area 1, compared to 62.67% (141/225) in area 2 and 44.44% (60/135) in area 3. This reflected the heterogeneity in village sizes between the different geographic areas of the programme: villages in area 1 were smaller than area 2 and 3, with respective median number of houses of 33 (IQR: 20–56), 65 (IQR: 42–120), and 96 (IQR: 53–160). 84.90% (641/755) of MP workers had received formal training from METF. However, 89.03% (487/547) of MPs were operated by at least one METF-trained MPW (Table 2). Among MPWs who has not received formal METF training, the majority had on the job training by either their MP supervisor or a previous MPW.

**Table 2:** Indicators of malaria post functioning collected during monitoring and evaluation visits

Indicator	Severity of dysfunction	Proportion	Percentage (%)
MP operated by at least 1 trained MP worker	Minor	487/547	89.03
Supervisor visit frequency in past 2 months	Minor		
0		141/534	26.40
1		87/534	16.29
2–3		121/534	22.66
> 4		185/534	34.64
Forms on site	Minor	541/547	98.90
Manual on site	Minor	531/548	96.90
Regular salary received	Minor	534/547	97.62
Another MP in village	N/A	112/547	20.48
Observed stockouts	Major		
ACT or RDT		44/551	7.99
ACT and RDT		3/551	0.54
Reported stockouts for > 2 days in the past month	Major	37/541	6.84
MP closure for > 24 h in the past 2 months	Major	144/549	26.23
Functionality category			
Major dysfunction		194/552	35.14
Minor dysfunction		186/552	33.70
No dysfunction		256/552	46.38
Insufficient data collected		23/552	4.17

At the time of M&E visit, 6.92% (38/549) of MPs did not have valid ACT medicines in stock, and 2.18% (12/551) did not have valid RDTs in stock. Overall this translates to 8% of MPs missing either ACT medicines or RDTs and 0.5% missing both ACT medicines and RDTs at the time of visit (Table 2).

Stockouts of either ACT medicines or RDTs for more than 2 days in the month prior to survey were reported in 6.84% (37/541) of MPs (Table 2), with twice as many stock outs of ACT medicines (6.92%) than RDTs (2.18%). Matching M&E data to weekly reports for those MPs with a stock out in the month prior to M&E visit revealed that the median stock levels of ACT medicines and RDTs were 120 tablets (IQR = 72–216) and 22 tests (IQR = 15–28) respectively. These levels were lower than for MPs without a reported stock out, with those MPs reporting median stock levels of 240 tablets of ACT medicines (IQR = 120–240) and 31 RDTs (IQR = 20–46).

A total of 35.14% of MPs were classified as having a major dysfunction at the time of M&E visit. However, when only considering the availability of diagnosis and treatment, 85.69% of MPs remained functional, that is, were stocked with ACT medicines and RDTs when the MP was open. Minor dysfunctions unlikely to disable the MPs ability to test and treat all malaria cases, accounted for 33.70% of MPs. MPs with no dysfunction, either major or minor, accounted for 46.38% of all MPs that had undergone a M&E visit.

### MP worker treatment knowledge

Treatment questionnaires were administered between January 2017 and August 2017 on a total of 722 occasions. From them the treatment questionnaire was administered once for 600 METF staff, and twice for 61 METF staff. The staff sample was made up of 635 (87.95%) MP workers, 79 (10.94%) MP supervisors, and 8 (1.11%) Zone and Assistant Zone-coordinators (see Table 4, Additional file 3).

### Quality control of rapid diagnostic tests

A total of 7109 RDTs conducted at MPs between December 2015 and December 2017 were rechecked at SMRU in Mae Sot at a median time of 91 days after being performed. From them 3703 (52.09%) RDTs were from area 1, 1829 (25.73%) from area 2, and 1577 from area 3 (22.18%). A total of 7031 (98.90%) of RDTs had a written record of test interpretation by MPW which were missing from 78 RDT only. Of the 7031 with written interpretation on the test, 5995 were recorded as negative, 380 as *P. falciparum* positive, 7 as mixed infection (*P. falciparum *+* P. vivax)*, 634 as *P. vivax* positive, 12 as invalid, and 3 had equivocal record (1 recorded “*P vivax*-negative” and 2 reported simply as “+ve”).

From the 387 *P. falciparum* positive and mixed infection positive RDTs, 349 (90.18%) were confirmed by the control as *P. falciparum* positive, and 15 (3.87%) were rechecked as *P. falciparum* negative, of which 2 were confirmed as *P. vivax* positive (*P. vivax* wrongly reported as *P. falciparum)*. From the 634 *P. vivax* positive RDTs as well as the 2 “+ve” results, 400 (62.89%) were confirmed by control as *P. vivax* positive, and 51 (8.02%) were rechecked as *P. vivax* negative, of which 1 was confirmed as *P. falciparum* positive (*P. falciparum* wrongly reported as *P. vivax)*.

Of the 5995 negative RDTs, only 1239 (20.67%) could be confirmed as being negative. Another 1858 (30.99%) *P. falciparum* test lines could be confirmed as negative but not the *P. vivax* line.

Approximately half (47.70%) of the negative RDTs could not be confirmed either due to blood back flow in 1994 (33.26%) RDTs or fading of the control line in 863 RDTs (14.40%).

False negatives found by control consisted of 24 (0.40%) invalid RDTs due to blood migration or clearance problems, 1 (0.02%) *P. vivax* positive RDT, and 2 (0.03%) *P. falciparum* positive RDTs.

Rather than following the manufacturer’s instructions to wait until the background had cleared within 30 min, a fixed reading time was commonly used (in 85% of RDTs). Reading times of < 10 min or > 30 min, or times unrecorded were rare (2.69%). The presence of dried blood remaining on the side of the well was found in 10.56% of RDTs, which could directly affect the sensitivity of the test.

In 4.50% of the tests the control line was very weak and was no longer visible in 13.50% of RDTs. Additionally, 5.42% of RDTs were dirty or damaged after use.

### Access to MP by population

In the MPs visited during M&E the average consultation rate was 4.1 consultations per week per 100 households (IQR = 0–5) in the 2 months prior to survey, but the average consultation rate by area was heterogenous. In area 1 where the highest burden of disease remains, the average consultation rate was higher with 6.4 (IQR = 0–8.3) consultations per week per 100 households, compared to 3.2 (IQR = 0.6–4) consultations per week per 100 households in area 2, and 2.4 (IQR = 0–3.2) per week per 100 households in area 3.

The delay between fever onset and consultation at a MP was between 0 and 48 h for 80.96% of consultations, between 2 and 3 days for 13.01% of consultations, and more than 3 days for 6.03% of all consultations. The time between fever onset and consultation was similar for all areas.

### Weekly reporting system and data completion

From April 2014 to November 2017, 140905 entries from 1242 MPs were included in the weekly database. From this database, the frequency of alerts previously defined (Table 1) was investigated (Table 4). The most commonly observed alert was late report (5.88%).

### Alert of MP dysfunction through weekly reporting

Weekly defined alerts of possible dysfunctions were associated with several problems observed by the M&E team. A weekly alert of no ACT medicines in stock was associated with an observed ACT medicines stockout (OR: 6.33, 95% CI 3.40, 11.79), but also no manual on site (OR: 5.49, 95% CI 2.42, 12.45), and the MPW reporting that they had not received a salary regularly (OR: 4.94, 95% CI 1.92, 12.72) (Table 5).

The alert for no RDTs was associated with increased odds of the MPW having no formal training (OR: 10.99, 95% CI 5.30, 22.77) and no manual on site (OR: 9.64, 95% CI 4.08, 22.79). Late weekly reports were associated with increased odds of no ACT medicines in stock at the time of M&E visit (OR: 2.12, 95% CI 1.27, 3.53), as well as a regular salary not being received (OR: 3.90, 95% CI 2.03, 7.46) (Table 5). No additional statistically significant associations were found. Additional file 4 gives the results for all univariate associations between M&E data and weekly defined alerts (see Additional file 4).

Univariate associations between M&E results and visit type revealed that there was an increase in the odds of a MP not having ACT medicines in stock in targeted visits compared to random visits (OR: 2.16, 95% CI 1.07, 4.38), and the odds of another MP operating in the village was higher in those selected MPs compared to random (OR: 1.88, 95% CI 1.17, 3.02). No additional statistically significant associations were found. Additional file 5 gives the results for all univariate associations between M&E results and visit type (see Additional file 5).

## Discussion

Access to diagnosis and treatment for all persons living within a malaria endemic region is essential in addressing malaria elimination targets. Operational since 2014, METF has set up and resourced over 1200 malaria posts to deliver uninterrupted access to community-based diagnosis and treatment of malaria in this region. In the 3 years of operation, this region has seen decreases in *P. falciparum* malaria incidence between 60 and 98% [10]. Over the same time period, following a rapid decline in malaria incidence between 2010 and 2014, decreases in confirmed malaria cases have slowed down and become more heterogenous across Myanmar [17]. In Kachin state, a similarly forested and mountainous region with hard-to-reach populations, increasing incidence was reported [18].

Ensuring the quality of care remains high is essential to the continuing decrease in malaria incidence in this region and the M&E of these MPs is a key component to METF programme operations.

### Malaria post worker performance

The METF programme invested strongly in its MPWs, through a long initial training (3 days, brought to 5 days to ensure more time for practice), refresher trainings, emphasis on regular supervision and data reporting, and provision of a financial compensation. The proportion of METF-trained MPWs remained high (84.90%), ensuring that 89.03% of MPs were operated by at least one METF-trained MPW (Table 2). Lack of formal METF training was frequently compensated by on the job training by their MP supervisor or a fellow MPW when they took over from a resigning MPW. Knowledge gaps could also be filled using guidance from the manual present in 96.90% of MP, from the MP supervisor or a neighbouring MPW.

MPW treatment knowledge was high overall, despite some special care cases having a lower proportion of correct responses (Table 3). The METF MPW algorithm included the treatment of all non-severe malaria cases, including children under 5 and pregnant women. This difference with other community-based programmes was necessary in this hard-to-reach setting where access to referral structures was particularly challenging.

**Table 3:** Proportions of correct and incorrect responses to the treatment questionnaire

Treatment quiz question	MP worker		MP supervisors, zone and assistant zone coordinators	
	Proportion	Percentage (%)	Proportion	Percentage (%)
1. Treatment for 1st trimester pregnancy *P. f*	533/635	83.94	74/87	85.06
2. Response to vomiting > 1 h after taking drug	270/635	42.52	49/87	56.32
3. Treatment for 2nd or 3rd trimester pregnancy *P. f*	301/635	47.40	44/87	50.57
4. Exclusion criteria for single low dose primaquine	441/635	69.45	72/87	82.76
5. Treatment for *P. f* if AL allergy	268/635	42.20	60/87	68.97
6. Variable used for drug dosage calculation	585/635	92.13	84/87	96.55
7. Correct treatment for mix infection (non-pregnant, aged > 5 months)	326/635	51.34	55/87	63.22
8. Correct treatment for breast-feeding mother *P. f*	318/635	50.08	49/87	56.32
9. Treatment for 6-month to 5-year old child *P. f*	330/635	51.97	47/87	54.02
10. Treatment for non-pregnant adult with *P. f* positive RDT and no fever	484/635	76.22	76/87	87.36
11. Treatment/conduct for patient with *P. f* who cannot eat or drink^a^	271/635	42.68	35/87	40.23
12. Treatment for *P. f* in child with fever, just woken from convulsion	383/635	60.31	53/87	60.92
13. Treatment of patient with *P. f* positive RDT, 1 week after complete malaria treatment	257/635	40.47	37/87	42.53
14. Treatment for *P. v* in child treated 1 month ago for *P. v*	467/635	73.54	60/87	68.97
15. Reason for treating *P. f* within 48 h of fever	367/635	57.80	70/87	80.46
16. Conduct if patient vomits drug < 30 min after taking it	479/635	75.43	67/87	77.01
17. Product to administer with AL to facilitate absorption	526/635	82.83	76/87	87.36
18. Treatment for patient 1st trimester pregnancy with *P. v*	383/635	60.31	60/87	68.97
19. Treatment of *P. f* in adult	494/635	77.80	73/87	83.91
20. Treatment for pregnant patient 1st trimester pregnancy with mixed infection (*P. f *+ *P. v)*	428/635	67.40	67/87	77.01

^a^55.17% of replies were to “refer immediately” (Additional file 3 details the proportions and percentages of responses to all questions)

RDT quality control showed that improvements can be made to the use of RDTs at the MPs. Delays in the reading of the RDTs occurred which could have increased the proportion of positive tests rechecked as negative due to the possible fading in a faint positive line. Blood backflow and the fading of signal with time impacted the ability to run quality control on some samples. Moreover, inappropriate storage of RDTs may explain the 4.50% of RDTs with a faded control line, 13.50% of RDTs with no visible control line, and 5.42% of RDTs that were damaged. Fewer positive RDTs for *P. vivax* than *P. falciparum* were confirmed by control which is explained by the *P. vivax* line being first affected by a potential blood backflow. Despite this, RDT quality control showed promising results with a limited number of false positives and negatives, and low numbers of mis-speciation.

Analysis of weekly data revealed that the percentage of late reports was relatively high (5.88%) while the occurrence of missing reports was relatively low (0.40%) (Table 4). The proportion of reports where there was incorrect use of RDTs and ACT medicines was low overall (0.88% and 0.95% respectively). However, this remains an important predictor of MP functioning where all fever cases should be tested and all individuals presenting with a positive RDT should be treated. However, it remains unclear whether unrecorded RDT use and ACT use is due to issues in testing and treatment, or in the recording of information. Therefore, this needs to be verified in order to gain a clearer picture of where processes could be improved by matching systematically the individual patient records with the weekly reports in order to detect discrepancies and incoherence. Additionally, information on pregnancy status, severity of cases, and deaths was not captured in the weekly reports, which would provide further information on the delivery of MP services. However, updated data collection sheets will allow for this data to be routinely collected.

**Table 4:** Occurrence of a priori defined alerts in weekly data

Alert	Proportion	Percentage (%)
Late report	8281/140905	5.88
Missing report	560/140905	0.40
No RDT stock	1474/140905	1.05
No ACT stock	1371/140905	0.97
Incorrect RDT use	1239/140905	0.88
Incorrect treatment use	1340/140905	0.95
Invalid RDTs not repeated	188/140905	0.13

### Availability of testing and treatment

Weekly recorded data revealed that 1.05% and 0.97% of MPs reported zero stocks of RDTs and ACT medicines, respectively (Table 4). Univariate logistic regression revealed associations between anomalies in the weekly reported data (delays, stock-outs) and dysfunctions observed during field visits (stock outs, but also lack of a manual, or irregular reception of salary). These univariate associations all point towards MP supervisor performance, where the collecting and reporting of data, and supplying the MP with ACT medicines and a regular salary, are all the responsibility of the MP supervisor (Table 5). No MP supervisor visit in the previous 2 months was recorded in 42.69% of the MPs (Table 2), however this did not mean no contact occurred during this period. Regular meetings between the MPWs and MP supervisors take place at central locations to transmit data and in some cases replenish supplies, but they may not be sufficient to routinely monitor activities which can only occur at the MP and often requires dedicated individual time.

**Table 5:** Univariate associations between monitoring and evaluation results and weekly alerts

M&E observed variable	Weekly alert	Odds ratio	95% CI	*P*-value
ACT not in stock	Report 0 ACT	6.33	3.40, 11.79	< 0.001
ACT not in stock	Late report	2.12	1.27, 3.53	0.004
Manual not on site	Report 0 ACT	5.49	2.42, 12.45	< 0.001
Manual not on site	Report 0 RDT	9.64	4.08, 22.79	< 0.001
Regular salary not received	Report 0 ACT	4.94	1.92, 12.72	0.001
Regular salary not received	Late report	3.90	2.03, 7.46	< 0.001
MPW not trained by METF	Report 0 RDT	10.99	5.30, 22.77	< 0.001

Increases in the frequency of MP supervisor visits would allow for improved stock monitoring and management resulting in fewer stockouts, reductions in late and missing reports, and improvements in the proportion of MPWs receiving a regular salary from their MP supervisor. Increasing the skills of MP supervisors to improve their ability to perform their duties (specifically in stock management) was also identified as a key intervention.

Previous studies have shown that improvements in MP supervisor presence can lead to an increase in healthcare facility performance, improved access to materials, and health workers receiving more feedback on their performance, resulting in increased motivation [19–21].

At the time of M&E visit there was more than three-times as many stockouts of ACT medicines (6.92%) than RDTs (2.18%). However, median stock levels recorded in weekly data revealed ACT medicines and RDTs were generally well maintained (120 tablets for ACT and 22 RDTs). This along with RDT and ACT medicine stock levels found in weekly data suggest that test and treatment availability are sufficient to address village needs. However, continuous monitoring and replenishment of these supplies is necessarily in order to decrease stockout occurrence. Moreover, the univariate logistic regression results suggest differences in the ability for weekly reported ACT medicine and RDT stock levels to predict stock levels at M&E visit. While associations between these variables across the weekly and M&E data might be expected, one does not inherently predict the other as replenishment of stocks could occur before M&E visit, or differences in stocks could suggest discrepancies in the weekly data reporting. While it is not clear which of these is true, the univariate logistic regression results suggest that weekly ACT medicines stocks more accurately predict ACT medicine stock outs during M&E visit, thus allowing for possible replenishment at that time, while the same is not true for RDT stocks across these MPs.

The average consultation rates remained high, especially in area 1 where the highest burden of disease remains. Moreover, the majority of consultations occur within the first 3 days of fever. This suggests that the majority of patients are presenting to a MP before becoming infectious, thereby contributing to the reduction of malaria transmission.

### Malaria post functionality

A total of 64.60% of MPs were classified as being open and fully stocked with ACT medicines and RDTs during M&E visits. Moreover, a total of 85.69% were stocked at the time of visit with no stockouts in the previous 2 months. Improvements to supervisor frequency may improve gaps in ACT medicine and RDT stock requirements and improve overall MP performance. Moreover, further training to enhance MPW knowledge in areas such as handling of malaria in the 2nd and 3rd trimester of pregnancy, treatment of *P. falciparum* in children, treatment in patients who cannot eat or drink, and treatment for those who remain *P. falciparum* positive 1 week following complete treatment, would ensure appropriate handling of complicated presentations when they occur. Additionally, training opportunities could be utilized to promote correct RDT use and storage.

## Conclusion

This report confirms the importance of monitoring and evaluation and how its outputs can inform programme operations and future areas of improvement. The MPs operating in the METF programme perform to a high standard, with the majority offering uninterrupted access to diagnosis and treatment, as indicated by the high proportion of fully-functioning MPs at time of M&E visit. Attendance to the MPs was also high, and the majority of patients presented to a MP within the first 3 days of fever, providing further evidence of the acceptability of the MP in the village setting and for its impact on malaria elimination in this region. This report also shows that the use of RDTs in the MP provide an effective method for diagnosis, with a low number of false negatives detected through RDT quality control. Areas of improvement as indicated in this report should focus on improved supervision of the MPs in order to strengthen ACT medicine and RDT stock replenishment, timely reporting of data, and ensuring a regular salary is provided to MPWs. Additionally, focusing on improving the knowledge of MPWs in some key areas of complicated case management as well as the reading of, and storage of RDTs will ensure the appropriate handling and diagnosis of these individuals when presenting to the MP as well allowing RDT quality control checks to be easily undertaken.

## Additional files

**Additional file 1.** Malaria Post assessment sheet used to collect information on malaria post activities during monitoring and evaluation visits.

**Additional file 2.** Treatment questionnaire administered to malaria post workers, malaria post supervisors, and zone and assistant zone coordinators.

**Additional file 3.** Summary of all response frequencies and percentages to treatment questionnaire.

**Additional file 4.** Complete univariate associations between monitoring and evaluation results and weekly alerts.

**Additional file 5.** Complete univariable associations between monitoring and evaluation results and M&E visit type (targeted or random).

## Abbreviations

EDT: early diagnosis and treatment
ACT: artemisinin-based combination therapy
RDT: rapid diagnostic test
MP: malaria post
MPW: malaria post worker
METF: malaria elimination task force
M&E: monitoring and evaluation
OR: odds ratio
CI: confidence interval
