# Supplementary material for: The role of monitoring and evaluation to ensure functional access to community-based early diagnosis and treatment in a malaria elimination programme in Eastern Myanmar
Source: Malar J. 2019 Feb 22;18:50. doi: 10.1186/s12936-019-2677-2 (PMC6387481; doi:10.1186/s12936-019-2677-2)
Supplement: Supplementary file 1 — Additional file 1. Malaria Post assessment sheet used to collect information on malaria post activities during monitoring and evaluation visits. [file 12936_2019_2677_MOESM1_ESM.docx]

**Additional file 1. Malaria Post Assessment Sheet.**

**Malaria Post Assessment**

Village Name___________________ Township___________ District ________State______

^[[1]](#footnote-1)^Malaria Post Code___________________ HH number ^[[2]](#footnote-2)^: ______________

Village GPS coordinates: LAT: ­­­­­­­­_________________ LONG: ____________________

| Name of ^[[3]](#footnote-3)^Malaria post worker  TRAINING: Yes No  RETRAINING: Yes No | | (1) ____________________ | (2) _____________________ |
| --- | --- | --- | --- |
|  |  | |  |
| Name of MP Supervisor __________________________________ | | | |
| MP worker not present | Number of days since MPW away: ……… Number of days until back: ………. | | |
| If not at post, where did the MPW go? | …………………………………………………… | |  |

**Assessment questions to MP workers (Ask to malaria workers directly)**

| 1 | Was the MP closed for > 24 hours in last 2 months?  If MPW was available even if MP was closed, mention in remark | Condition | **Comment/remark** |
| --- | --- | --- | --- |
|  |  | YES NO |  |
| 2 | Are there valid ACTs in the MP? | YES NO |  |
| 3 | Are there valid RDTs in the MP? | YES NO |  |
| 4 | Were there >2 days out of stocks (RDTs or ACTs) in the past 4 weeks?  -----------------------------------------------------------------------------------  Adequate or sufficient medication and supplies (observe and check carefully)  SD biolines = tests ACT= tabs CQ = tabs PMQ = tabs  Clindamycin | YES NO | If no, ask why |
| 5 | How are the results reported?  SMS Paper Other (……………….) |  |  |
| 6 | Does the MPW receive regular financial incentive? | YES NO |  |
| 7 | Is there another MP in the village? | YES NO |  |
| 8 | If YES, specify the supporting organization |  | |
| 8b | If Yes, do you receive malaria data from them? | YES NO | |
| 9 | How often did you receive the visit of your MP supervisor in the last 2 months? …………………… time(s) | 1 Per Month  <1 Per Month  >1 Per Month |  |

**Assessment by Evaluator (Check - List)**

| 1 | Is there a Malaria Post Manual in the MP? | YES NO |
| --- | --- | --- |
| 2 | Are there reporting forms in the MP? | YES NO |
| 3 | Is there a logbook (daily recording of individual patients) in the MP? | YES NO |
| 4 | Are the “Days of fever” recorded for each patient? (Review the daily record sheets) | YES NO |
| 5 | Are there more than 5 consecutive days without activity^[[4]](#footnote-4)^ in the logbook? | YES NO |

1. Comment or suggestions from malaria post worker.

_________________________________________________________________________________________________________________________________________________________________________________________________________________________________________________________________________________

1. Comments or suggestion from the observer.

_________________________________________________________________________________________________________________________________________________________________________________________________________________________________________________________________________________

| Name: _________________________  Signature: _____________________ | |
| --- | --- |
| Date: _________________________ |  |

1. Malaria Post [↑](#footnote-ref-1)
2. House that are inhabited [↑](#footnote-ref-2)
3. Malaria Post Worker [↑](#footnote-ref-3)
4. Activity = (case of fever)

   METF Monitoring and Evaluation Form (ENG) V5.0 – 09 Jan 2017 Page 2 [↑](#footnote-ref-4)
