# Supplementary material for: The role of monitoring and evaluation to ensure functional access to community-based early diagnosis and treatment in a malaria elimination programme in Eastern Myanmar
Source: Malar J. 2019 Feb 22;18:50. doi: 10.1186/s12936-019-2677-2 (PMC6387481; doi:10.1186/s12936-019-2677-2)

**Additional file 2**

**. Treatment questionnaire administered to malaria post workers, malaria post supervisors, and zone and assistant zone coordinators.**

| 1. | What is the correct treatment for pregnant patient diagnosed with *P. falciparum* during 1^st^ trimester of pregnancy?   1. Quinine + Clindamycin x 7 days (Q7C7) 2. Co-artem + Primaquine (COA3 + PQ 1 time) 3. Co-artem + Primaquine (COA3 + PQ 3 time) 4. Chloroquine (CQ3) | |  |
| --- | --- | --- | --- |
|  |  |  |  |
| 2. | What to do if a patient vomits the drug more than 1 hour after taking it?   1. Repeat the 1^st^ dose again. 2. Don't need to repeat the 1^st^ dose 3. Stop the treatment 4. Change the treatment | |  |
| 3.   | What is the correct treatment for pregnant patient diagnosed with *P. falciparum* during and 3^rd^ trimester of pregnancy (between 3 — 9 months)?   1. Co-artem + Primaquine (COA3 + PQ 1 time) 2. Co-artem only (COA3) 3. Chloroquine (CQ3) 4. Quinine + Clindamycin x 7 days (Q7C7) | |  |
| 4.   | Primaquine should be given to   1. f trimester of pregnancy 2. 2^nd^ trimester of pregnancy 3. Breastfeeding mother with child less than 6 months. 4. None of above is true | |  |
| 5. | Treatment of *P. falciparum* in a patient who is allergic to coartem?   1. Quinine + Clindamycin x 7 days (Q7C7) 2. Chloroquine 3. Primaquine 4. No treatment | |  |
| 6. | What are anti-malaria drug dosage calculations based on   1. Weight (kilogram — kg) 2. Weight (pounds — lbs) 3. Age (months) 4. Age (years) | |  |
|  |  |  |  |
| 7. | What is the correct treatment for a non-pregnant patient (age > 5 months) diagnosed with mixed infection (*P. falciparum* & *P. vivax*).   1. Co-artem + Primaquine (COA3 + PQ 3 time) 2. Chloroquine (CQ3) 3. Quinine + Clindamycin x 7 days (Q7C7) 4. Co-artem + Primaquine (COA3 + PQ 1 time) | |  |
| 8.   | What is the correct treatment for breast-feeding mother with 5 months old child diagnosed with  *P. falciparum*?   1. Quinine + Clindamycin x 7 days (Q7C7) 2. Co-artem + Primaquine (COA3 + PQ 1 time) 3. Co-artem + Primaquine (COA3 + PQ 3 time) 4. Co-artem only | |  |
| 9. | Treatment of *P. falciparum* in a child under 5 years (but > 6 months old)   1. Co-artem + Primaquine (COA3 + PQ 1 time) 2. Chloroquine (CQ3) 3. Quinine + Clindamycin x 7 days (Q7C7) 4. Co-artem only | |  |
| 10 | How will you treat a non-pregnant adult patient with no fever and *P. falciparum* RDT positive?   1. Quinine + Clindamycin x 7 days (Q7C7) 2. Chloroquine 3. Co-artem + Primaquine (COA3 + PQ 1 time) 4. No treatment till fever develops | |  |
| 11 | Treatment of *P. falciparum* in a patient that cannot eat or drink  a) Refer the patient to nearest hospital or health centre  b) Try to give 1^st^ dose of Co-artem and refer quickly to nearest hospital or health centre  c)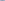 Give Co-artem and primaquine (COA3 + PQ) and send back home  d) No treatment | |  |
| 12 | Treatment of *P. falciparum* in child with fever who just wake up from a convulsion   1. Cool the child and try to give 1^st^ dose of Co-artem and refer 2. No treatment 3. Refer the patient to hospital or health centre without treatment 4. Give Co-artem and primaquine (COA3 + PQ) and send back home | |  |
| 13 | Treatment of patient with a positive *P. falciparum* RDT 1 week after a complete treatment for malaria?   1. Quinine + Clindamycin x 7 days (Q7C7) 2. Chloroquine 3. Repeat Co-artem 4. Give paracetamol and ask to come back if fever continues | |  |
| 14 | Treatment of *P. vivax* in a child who was treated 1 month ago for *P. vivax*?   1. Chloroquine 2. Co-artem 3. Primaquine 4. No treatment | |  |
| 15 | Why do we need to treat *P. falciparum* within 48 hours of fever?   1. To stop the transmission of *P. falciparum* from one person to another 2. To stop the spread of malaria drug resistance in the area 3. To prevent the patient to become severe. 4. All of the above is true | |  |
| 16   | What to do if a patient vomits the drug less than 30 min after taking it?   1. Repeat the 1^st^ dose again. 2. Don't need to repeat the 1^st^ dose 3. Stop the treatment 4. Change the ACTs | |  |
| 17 | | How do we administer Coartem for better absorption?   1. Water 2. Some fat (Milk or after meal) 3. Tea 4. Coffee |  |
| 18 | | What is the correct treatment for pregnant patient diagnosed with *P. vivax* in first trimester?   1. Co-artem 2. Chloroquine (CQ3) 3. Chloroquine + Primaquine (CQ3 + PQ) 4. Quinine + Clindamycin x 7 days (Q7C7) |  |
| 19 | | Treatment of *P. falciparum* in adult   1. Chloroquine (CQ3) 2. Quinine + Clindamycin x 7 days (Q7C7) 3. Co-artem only 4. Co-artem + Primaquine (COA3 + PQ 1 time) |  |
| 20 | | 2What is the correct treatment for pregnant patient diagnosed with Mixed infection (*P. falciparum* & *P. vivax)*  during 1^st^ trimester of pregnancy (within first 3 months)?   1. Co-artem + Primaquine (COA3 + PQ 3 time) 2. Chloroquine (CQ3) 3. Quinine + Clindamycin x 7 days (Q7C7) 4. Co-artem + Primaquine (COA3 + PQ 1 time) |  |


Supplement: Supplementary file 2 — Additional file 2. Treatment questionnaire administered to malaria post workers, malaria post supervisors, and zone and assistant zone coordinators. [file 12936_2019_2677_MOESM2_ESM.docx]
