# Supplementary material for: The role of monitoring and evaluation to ensure functional access to community-based early diagnosis and treatment in a malaria elimination programme in Eastern Myanmar
Source: Malar J. 2019 Feb 22;18:50. doi: 10.1186/s12936-019-2677-2 (PMC6387481; doi:10.1186/s12936-019-2677-2)
Supplement: Supplementary file 3 — Additional file 3. Summary of all response frequencies and percentages to treatment questionnaire. [file 12936_2019_2677_MOESM3_ESM.docx]

**Additional file 3. Summary of all response frequencies and percentages to treatment questionnaire.**

| **Treatment quiz question** | **MP worker** | | **MP supervisors, zone and assistant zone coordinators** | |
| --- | --- | --- | --- | --- |
|  | **Proportion** | **Percentage (%)** | **Proportion** | **Percentage (%)** |
| 1. Treatment for 1^st^ trimester pregnancy *P. f*   A – *correct response*  B  C  D | 533/635  39/635  20/635  43/635 | 83.94  6.14  3.15  6.77 | 74/87  5/87  4/87  4/87 | 85.06  5.75  4.60  4.60 |
| 1. Response to vomiting >1hr after taking drug   A  B – *correct response*  C  D | 201/635  270/635  112/635  52/635 | 31.65  42.52  17.64  8.19 | 16/87  49/87  18/87  4/87 | 18.39  56.32  20.69  4.60 |
| 1. Treatment for 2^nd^ or 3^rd^ trimester pregnancy *P. f*   A  B – *correct response*  C  D | 76/635  301/635  57/635  201/635 | 11.97  47.40  8.98  31.65 | 17/87  44/87  3/87  23/87 | 19.54  50.57  3.45  26.44 |
| 1. Exclusion criteria for single low dose primaquine   A  B  C  D– *correct response* | 51/635  77/635  66/635  441/635 | 8.03  12.13  10.39  69.45 | 4//87  8/87  3/87  72/87 | 4.60  9.20  3.45  82.76 |
| 1. Treatment for *P. f* if AL allergy   A – *correct response*  B  C  D | 268/635  98/635  124/635  145/635 | 42.40  15.43  19.53  22.83 | 60/87  9/87  9/87  9/87 | 68.97  10.34  10.34  10.34 |
| 1. Variable used for drug dosage calculation   A – *correct response*  B  C  D | 585/635  21/635  5/635  24/635 | 92.13  3.31  0.79  3.78 | 84//87  2/87  0/87  1/87 | 96.55  2.30  0  1.15 |
| 1. Correct treatment for mix infection (non-pregnant, aged >5months)   A  B  C  D – *correct response* | 144/635  84/635  81/635  326/635 | 22.68  13.23  12.76  51.34 | 14/87  2/87  16/87  55/87 | 16.09  2.30  18.39  63.22 |
| 1. Correct treatment for breast-feeding mother *P. f*   A  B  C  D – correct response | 201/635  81/635  35/635  318/635 | 31.65  12.76  5.51  50.08 | 24/87  13/87  1/87  49/87 | 27.59  14.49  1.15  56.32 |
| 1. Treatment for 6-month to 5-year old child *P. f*   A – *correct response*  B  C  D | 330/635  70/635  64/635  171/635 | 51.97  11.02  10.08  26.93 | 47/87  4/87  8/87  28/87 | 54.02  4.60  9.20  32.18 |
| 1. Treatment for non-pregnant adult with *P. f* positive RDT and no fever   A  B  C – *correct response*  D | 40/635  30/635  484/635  81/635 | 6.30  4.72  76.22  12.76 | 3/87  1/87  76/87  7/87 | 3.45  1.15  87.36  8.05 |
| 1. Treatment/conduct for patient with *P. f* who cannot eat or drink   A  B – *correct response*  C  D | 328/635  271/635  28/635  8/635 | 51.65  42.68  4.41  1.26 | 48/87  35/87  3/87  1/87 | 55.17  40.23  3.45  1.15 |
| 1. Treatment for *P. f* in child with fever, just woken from convulsion   A – *correct response*  B  C  D | 383/635  11/635  171/635  70/635 | 60.31  1.73  26.93  11.02 | 53/87  2/87  23/87  9/87 | 60.92  2.30  26.44  10.34 |
| 1. Treatment of patient with *P. f* positive RDT, 1 week after complete malaria treatment   A  B  C  D – *correct response* | 90/635  50/635  238/635  257/635 | 14.17  7.87  37.48  40.47 | 7/87  3/87  37/87  40/87 | 8.05  3.45  42.53  45.98 |
| 1. Treatment for *P. v* in child treated 1 month ago for *P. v*   A – *correct response*  B  C  D | 467/635  55/635  50/635  63/635 | 73.54  8.66  7.87  9.92 | 60/87  7/87  9/87  11/87 | 68.97  8.05  10.34  12.64 |
| 1. Reason for treating *P. f* within 48 hours of fever   A  B  C  D – *correct response* | 116/635  34/635  118/635  367/635 | 18.27  5.35  18.58  57.80 | 12/87  2/87  3/87  70/87 | 13.79  2.30  3.45  80.46 |
| 1. Conduct if patient vomits drug < 30min after taking it   A – *correct response*  B  C  D | 479/635  91/635  20/635  45/635 | 75.43  14.33  3.15  7.09 | 67/87  11/87  3/87  6/87 | 77.01  12.64  3.45  6.90 |
| 1. Product to administer with AL to facilitate absorption   A  B – *correct response*  C  D | 94/635  526/635  5/635  10/635 | 14.80  82.83  0.79  1.57 | 10/87  76/87  1/87  0/87 | 11.49  87.36  1.15  0 |
| 1. Treatment for patient 1^st^ trimester pregnancy with *P. v*   A  B – *correct response*  C  D | 45/635  383/635  47/635  160/635 | 7.09  60.31  7.40  25.20 | 2/87  60/87  2/87  23/87 | 2.30  68.97  2.30  26.44 |
| 1. Treatment of *P. f* in adult   A  B  C  D *– correct response* | 31/635  43/635  67/635  494/635 | 4.88  6.77  10.55  77.80 | 4/87  4/87  6/87  73/87 | 4.60  4.60  6.90  83.91 |
| 1. Treatment for pregnant patient 1^st^ trimester pregnancy with mixed infection (*P. f* + *P. v)*   A  B  C *– correct response*  D | 68/635  59/635  428/635  80/635 | 10.71  9.29  67.40  12.60 | 3/87  7/87  67/87  10/87 | 3.45  8.05  77.01  11.49 |
