# Supplementary material for: The role of monitoring and evaluation to ensure functional access to community-based early diagnosis and treatment in a malaria elimination programme in Eastern Myanmar
Source: Malar J. 2019 Feb 22;18:50. doi: 10.1186/s12936-019-2677-2 (PMC6387481; doi:10.1186/s12936-019-2677-2)
Supplement: Supplementary file 4 — Additional file 4. Complete univariate associations between monitoring and evaluation results and weekly alerts. [file 12936_2019_2677_MOESM4_ESM.docx]

**Additional file 4. Complete univariate associations between monitoring and evaluation results and weekly alerts.**

| **M&E measured variable** | **Weekly alert** | **Odds ratio** | **95% CI** | **p-value** |
| --- | --- | --- | --- | --- |
| MP not operated by trained MPW | Late report | 1.32 | 0.81, 2.17 | 0.266 |
|  | Missing report | 1.00 | - | - |
|  | No RDT stocks | 10.99 | 5.30, 22.77 | <0.001 |
|  | No ACT stocks | 1.89 | 0.91, 3.93 | 0.087 |
|  | Incorrect RDT use | 0.74 | 0.22, 2.42 | 0.616 |
|  | Incorrect treatment use | 1.51 | 0.67, 3.39 | 0.322 |
|  | Invalid RDTs not repeated | 5.5 | 0.28, 107.15 | 0.261 |
| MP closure for >24 hours in the past 2 months | Late report | 1.11 | 0.76, 1.62 | 0.589 |
|  | Missing report | 1.70 | 0.41, 7.14 | 0.467 |
|  | No RDT stocks | 0.54 | 0.21, 1.42 | 0.213 |
|  | No ACT stocks | 1.25 | 0.68, 2.31 | 0.468 |
|  | Incorrect RDT use | 1.10 | 0.52, 2.27 | 0.815 |
|  | Incorrect treatment use | 0.89 | 0.45, 1.76 | 0.737 |
|  | Invalid RDTs not repeated | 1.42 | 0.20, 9.82 | 0.724 |
| Observed ACT stock out | Late report | 2.12 | 1.27, 3.53 | 0.004 |
|  | Missing report | 1.00 | - | - |
|  | No RDT stocks | 1.00 | - | - |
|  | No ACT stocks | 6.33 | 3.40, 11.79 | <0.001 |
|  | Incorrect RDT use | 1.63 | 0.57, 4.62 | 0.360 |
|  | Incorrect treatment use | 0.93 | 0.29, 3.02 | 0.906 |
|  | Invalid RDTs not repeated | 1.00 | - | - |
| Observed RDT stock out | Late report | 0.98 | 0.30, 3.12 | 0.968 |
|  | Missing report | 1.00 | - | - |
|  | No RDT stocks | 1.48 | 0.20, 10.97 | 0.701 |
|  | No ACT stocks | 1.90 | 0.45, 7.95 | 0.378 |
|  | Incorrect RDT use | 1.23 | 0.17, 9.08 | 0.837 |
|  | Incorrect treatment use | 0.98 | 0.13, 7.21 | 0.987 |
|  | Invalid RDTs not repeated | 1.00 | - | - |
| Reported stock outs for >2 days in the past month | Late report | 1.66 | 0.94, 2.93 | 0.079 |
|  | Missing report | 2.00 | 0.24, 16.31 | 0.517 |
|  | No RDT stocks | 1.56 | 0.47, 5.17 | 0.468 |
|  | No ACT stocks | 1.67 | 0.66, 4.27 | 0.279 |
|  | Incorrect RDT use | 0.80 | 0.19, 3.33 | 0.756 |
|  | Incorrect treatment use | 0.97 | 0.30, 3.16 | 0.966 |
|  | Invalid RDTs not repeated | 1.00 | - | - |
| Regular salary not received | Late report | 3.90 | 2.04, 7.46 | <0.001 |
|  | Missing report | 6.02 | 0.73, 49.38 | 0.095 |
|  | No RDT stocks | 1.40 | 0.19, 10.34 | 0.743 |
|  | No ACT stocks | 4.94 | 1.92, 12.72 | 0.001 |
|  | Incorrect RDT use | 2.42 | 0.57, 10.18 | 0.229 |
|  | Incorrect treatment use | 0.93 | 0.13, 6.80 | 0.942 |
|  | Invalid RDTs not repeated | 1.00 | - | - |
| Forms not onsite | Late report | 1.00 | - | - |
|  | Missing report | 1.00 | - | - |
|  | No RDT stocks | 1.00 | - | - |
|  | No ACT stocks | 1.00 | - | - |
|  | Incorrect RDT use | 1.00 | - | - |
|  | Incorrect treatment use | 2.02 | 0.27, 14.93 | 0.492 |
|  | Invalid RDTs not repeated | 1.00 | - | - |
| Manual not onsite | Late report | 1.18 | 0.48, 2.94 | 0.716 |
|  | Missing report | 1.00 | - | - |
|  | No RDT stocks | 9.64 | 4.08, 22.79 | <0.001 |
|  | No ACT stocks | 5.49 | 2.42, 12.45 | <0.001 |
|  | Incorrect RDT use | 1.00 | - | - |
|  | Incorrect treatment use | 0.70 | 0.09, 5.11 | 0.724 |
|  | Invalid RDTs not repeated | 1.00 | - | - |
