# Supplementary material for: The role of monitoring and evaluation to ensure functional access to community-based early diagnosis and treatment in a malaria elimination programme in Eastern Myanmar
Source: Malar J. 2019 Feb 22;18:50. doi: 10.1186/s12936-019-2677-2 (PMC6387481; doi:10.1186/s12936-019-2677-2)
Supplement: Supplementary file 5 — Additional file 5. Complete univariable associations between monitoring and evaluation results and M&E visit type (targeted or random). [file 12936_2019_2677_MOESM5_ESM.docx]

**Additional file 5. Complete univariable associations between monitoring and evaluation results and M&E visit type (targeted or random).**

| **M&E measured variable** | **Odds ratio** | **95% CI** | **p-value** |
| --- | --- | --- | --- |
| MP not operated by trained MPW | 1.79 | 0.98, 3.25 | 0.055 |
| MP closure for >24 hours in the past 2 months | 0.72 | 0.44, 1.18 | 0.197 |
| Observed ACT stock out | 2.16 | 1.07, 4.38 | 0.032 |
| Observed RDT stock out | 0.78 | 0.17, 3.61 | 0.751 |
| Reported stock outs for >2 days in the past month | 1.28 | 0.59, 2.80 | 0.533 |
| Regular salary not received | 0.32 | 0.04, 2.47 | 0.273 |
| Forms not onsite | 1.96 | 0.35, 10.83 | 0.441 |
| Manual not onsite | 2.19 | 0.79, 6.05 | 0.132 |
